# Detecting Disease in Radiographs with Intuitive Confidence

**DOI:** 10.1155/2015/946793

**Published:** 2015-10-01

**Authors:** Stefan Jaeger

**Affiliations:** U.S. National Library of Medicine, National Institutes of Health, 8600 Rockville Pike, Bethesda, MD 20894, USA

## Abstract

This paper argues in favor of a specific type of confidence for use in computer-aided diagnosis and disease classification, namely, sine/cosine values of angles represented by points on the unit circle. The paper shows how this confidence is motivated by Chinese medicine and how sine/cosine values are directly related with the two forces Yin and Yang. The angle for which sine and cosine are equal (45°) represents the state of equilibrium between Yin and Yang, which is a state of nonduality that indicates neither normality nor abnormality in terms of disease classification. The paper claims that the proposed confidence is intuitive and can be readily understood by physicians. The paper underpins this thesis with theoretical results in neural signal processing, stating that a sine/cosine relationship between the actual input signal and the perceived (learned) input is key to neural learning processes. As a practical example, the paper shows how to use the proposed confidence values to highlight manifestations of tuberculosis in frontal chest X-rays.

## 1. Introduction

With increasing performance of automated disease detection, computer-aided diagnosis (CAD) is becoming a serious alternative to the established diagnostic workflow [1]. CAD can lead to better diagnostics by providing physicians with critical information extracted from many relevant cases through machine learning techniques. However, the communication between man and machine should be intuitive so that a physician can readily use the machine output for diagnostics. This paper describes a method for generating confidence values for local manifestations of tuberculosis detected automatically in a frontal chest X-ray (CXR). The proposed confidence values are motivated by Chinese medicine and neural signal processing. The higher the confidence the more likely it is that the detected abnormal region is indeed abnormal. The confidence values correspond to sine or cosine and can thus be represented by a point on the unit circle. A confidence of sin(45°) describes a region that the machine considers neither normal nor abnormal. This confidence therefore describes a state of equilibrium where Yin and Yang are in perfect balance, a state of nonduality. Note that this is in contrast to the conventional understanding in traditional Chinese medicine, where the equilibrium between Yin and Yang denotes a healthy state. However, in the author's opinion this is incorrect because the state of equilibrium between Yin and Yang is the state of nonduality, but assigning attributes, such as “healthy state,” clearly introduces duality. Therefore, the equilibrium of Yin and Yang should be associated with neither normality nor abnormality. The proposed confidence values meet this requirement. The state of equilibrium corresponds to a state of uncertainty, in which it is entirely unclear whether a lung region is normal or abnormal. This could be because there is no indication for one or the other, or there is supporting evidence in equal shares for both normality and abnormality.

A second motivation of the confidence values proposed here is that they are designed to be intuitively accessible by a radiologist, in the sense that they can be easily learned without much calibration on the part of the radiologist. The proposed confidence values should represent similar numerical values a human expert would provide if asked to quantify his confidence in a specific abnormality. This would guarantee that the opinion of the machine is put on an equal footing with the opinion of the radiologist. The radiologist can then integrate his own confidence with the machine confidence and reach the final verdict about whether a specific lung region should be considered normal or abnormal. The paper will resort to principles of neural signal processing to argue that the proposed confidence is intuitive because it forms the basic of synaptic learning.

The paper is structured as follows. In Section 2, the formal definition of Yin and Yang will be presented and the connection between the proposed confidence and the forces of Yin and Yang will be discussed in more detail. This chapter is largely motivated by earlier work in [2, 3]. In Section 3, the proposed confidence values will be used to represent manifestations of tuberculosis in frontal chest X-rays as a saliency map. Finally, a conclusion will summarize the main results.

## 2. Materials and Methods

Section 2.1 summarizes briefly the basic principles and processes of neural signal transduction that we can observe at chemical synapses.  Section 2.2 explains the information-theoretic model that is used in this paper to formalize the information flow occurring at a synapse, similar to [3], and shows why the proposed model makes sense given what is known today about neural signal processing. Following this introduction of the theoretical model, Section 2.3 shows a direct connection to Yin and Yang.  Section 2.4 then describes how synaptic learning is explained in the proposed information-theoretic model. Finally, Section 2.5 shows how these results motivate the proposed confidence values.

### 2.1. Neural Signal Transduction

This section presents a brief overview of the basic signal transduction principles, as they are needed for understanding the remainder of this paper. First, neurons and synapses are explained and then the Hodgkin and Huxley model describing electrical signal processing of nerve cells is presented. Most of the information in this subsection is taken from [3].

#### 2.1.1. Neurons and Synapses

**Figure 1:**
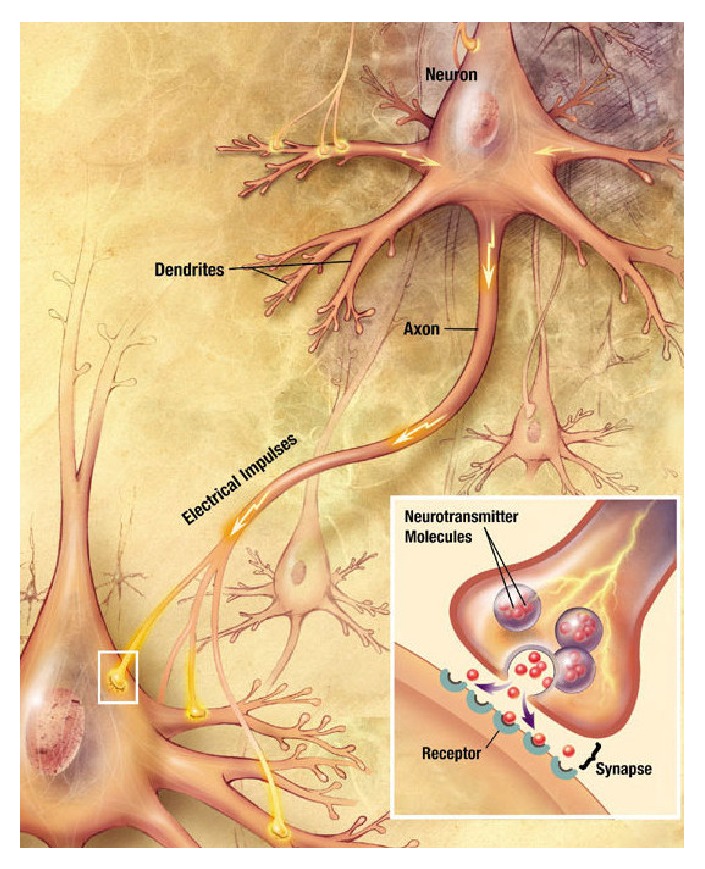
A signal propagating down an axon to the cell body and dendrites of the next cell (Source: NIA/NIH).

The human nervous system is composed of nerve cells, so-called neurons, which can communicate with each other through synapses. A synapse is a membrane-to-membrane junction that allows either chemical or electrical signal transmission. In the case of chemical synapses, which will be in the focus here, signals are transmitted via neurotransmitters that can bridge the synaptic cleft, a small gap between the membranes of two nerve cells. As an illustration, the diagram in Figure 1 shows two communicating neurons. A neuron can send a signal to another neuron through its axon, which is a protrusion with potentially thousands of synapses and which can extend to other neurons in distant parts of the body. A neuron can receive the signal via its soma or its dendrites that conduct the received signal to the cell body (see Figure 1). In both cases, the signal needs to pass a synapse that transmits the signal by molecular means, via neurotransmitters, through the synaptic cleft, from the presynaptic terminal to the postsynaptic terminal. The small volume of the synaptic cleft allows neurotransmitter concentration to increase and decrease rapidly. Prior to any signal transmission, the neurotransmitters are enclosed in small spheres, synaptic vesicles, at the presynaptic terminal. On the other side, the postsynaptic terminal provides receptors for neurotransmitters traveling through the synaptic cleft. The lower right corner of Figure 1 shows a close-up of a synapse. The adult human brain contains between 10^14^ and 5 × 10^14^ of these synapses. Synapses, and the way they transmit information, are crucial to the biological computations that underlie perception and thought. The common understanding is that synapses, and changes in their behavior, are responsible for memorization and human learning. To get insight into these processes, it is essential to study the molecular processes underlying signal transmission.

**Figure 2:**
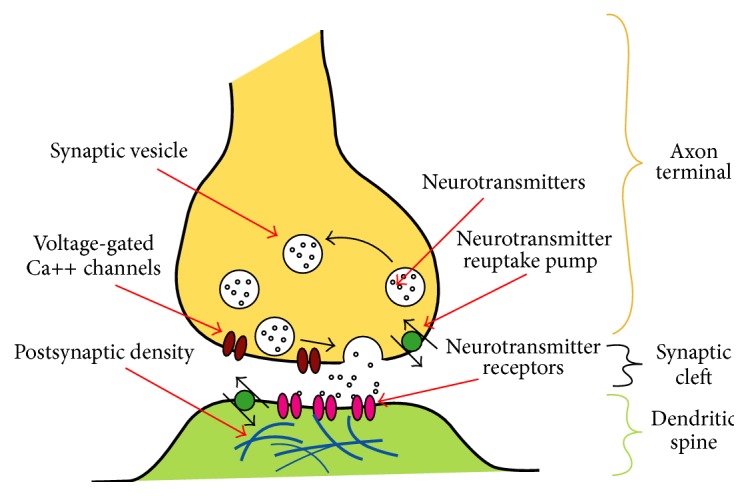
Signal transmission at a chemical synapse [21] (Source: Wikipedia, Surachit, Nrets).

Signal transmission at a chemical synapse is a multistep process (see Figure 2). The transmission is triggered by an electrochemical excitation (action potential) at the presynaptic terminal. The excitation causes calcium channels to open, allowing calcium ions to flow into the presynaptic terminal. The increased concentration of calcium ions in the presynaptic terminal causes the vesicles to release their neurotransmitters into the synaptic cleft. Some of these neurotransmitters bind to the receptors of the postsynaptic terminal, which opens ion channels in the postsynaptic membrane, allowing ions to flow into or out of the postsynaptic cell. This changes the transmembrane potential, leading to an excitation or inhibition of the postsynaptic cell. In this way, the action potential from the presynaptic terminal has created a postsynaptic potential by molecular means. Eventually, the docked neurotransmitters will break away from the postsynaptic receptors. Some of them will be reabsorbed by the presynaptic cell to initiate another transmission cycle.

#### 2.1.2. Model by Hodgkin and Huxley

In 1952, in a seminal paper, Hodgkin and Huxley proposed a set of equations explaining the electrical characteristics of nerve cells and their underlying ionic mechanisms [4]. Their entry point is the sodium conductance at the cell membrane of a nerve cell. Similar to calcium ions, sodium ions are largely responsible for generating action potentials in nerve cells. A nerve cell membrane has voltage-gated ion channels that are shut when the membrane is close to the resting potential. Once the membrane potential increases to a critical value, these ion channels open and allow sodium ions to pass the cell membrane and travel into the cell. The influx of sodium ions increases the membrane potential even more, causing more ion channels to open and thus allowing more sodium ions to move into the cell. This reinforcing process stops once the membrane potential has reversed and the nerve cell has reached its action potential. After reaching the action potential, the sodium channels close rapidly, preventing any more sodium ions from entering the cell. The sodium ions are then transported out of the nerve cell and the cell returns to its resting potential. Understanding the temporal change of the sodium concentration is therefore important for understanding the generation and transportation of action potentials.

In their mathematical model, Hodgkin and Huxley assume that the sodium conductance is proportional to the number of specific molecules on the inside of the membrane but that the conductance is independent of the number of molecules on the outside [4]. According to Boltzmann's principle the proportion *P*_*i*_ of the molecules on the inside of the membrane is related to the proportion *P*_*o*_ on the outside by

(1) $$\begin{array}{c} \frac{P_{i}}{P_{o}}=\exp\left[\frac{\left(w+zeE\right)}{kT}\right], \end{array}$$

where *E* is the potential difference between the outside and the inside of the membrane, *w* is the work required to move a molecule from the inside to the outside of the membrane when *E* = 0, *e* is the absolute value of the electronic charge, *z* is the valency of the molecule (i.e., the number of positive electronic charges on it), *k* is Boltzmann's constant, and *T* is the absolute temperature [4]. With *P*_*i*_ + *P*_*o*_ = 1, the expression for *P*_*i*_ becomes

(2) $$\begin{array}{c} P_{i}=\frac{1}{1+\exp\left(-\left(w+zeE\right)/kT\right)}. \end{array}$$

The concentration of the molecules on the inside of the membrane thus follows a sigmoid function, which will become important later in this paper.

### 2.2. Information-Theoretic Model

The formal derivation of the theoretical information processing model proposed here begins with a closer look at the calcium ion concentration in the synaptic cleft close to the postsynaptic terminal. Let this concentration be *p*_*i*_, as opposed to the outer concentration in the presynaptic terminal *p*_*o*_. Then, let us assume that the strength *S* of the stimulus arriving at the presynaptic terminal is determined by the ratio of outer to inner calcium ion concentration *p*_*o*_/*p*_*i*_, or

(3) $$\begin{array}{c} S=\frac{1-p_{i}}{p_{i}}. \end{array}$$

For example, for an inactive synapse, the concentration of calcium ions in the synaptic cleft will be one, that is, *p*_*i*_ = 1, as there will be no calcium ions in the presynaptic terminal. Consequently, according to (3), the signal strength is zero. On the other hand, for an active synapse, calcium ions can freely flow into the presynaptic terminal until a concentration equilibrium is reached between calcium ions in the presynaptic terminal and calcium ions in the synaptic cleft, which means *p*_*i*_ = *p*_*o*_ = 0.5. In this case, according to (3), the signal strength *S* is maximum; that is, *S* = 1.

Now, let us assume that the postsynaptic terminal performs a linear learning function, taking the strength of the input signal as input. Describing synaptic learning by a linear function is not uncommon. In fact, there are reasons to believe that this reflects the biological reality, and there have been many approaches in machine learning that model synaptic learning with linear functions [5–8]. The characteristic feature of the linear learning function presented here is that it operates on the information content of the input signal rather than on the signal itself. To do so, it uses the standard dual logarithm to measure information, as investigated by Shannon in his seminal paper [9]. The following equation describes this linear relationship [3]:

(4) $$\begin{array}{c} I=-m\cdot \log_{2}\left(\frac{1-p_{i}}{p_{i}}\right)+c, \end{array}$$

where *I* is the information learned by the linear model at the postsynaptic terminal. The two parameters that affect learning here are the slope *m* and the offset *c* of the linear model.

One of the main motivations of using this model is the form the equation of *p*_*i*_ assumes when we resolve (4) for *p*_*i*_:

(5) $$\begin{array}{c} p_{i}=\frac{1}{1+\exp\left(-\left(\left(I-c\right)\cdot \ln\left(2\right)\right)/m\right)}. \end{array}$$

Note that the dual logarithm has been converted into the natural logarithm in (5). This produces the same type of sigmoid function that was used in Section 2.1.2 to describe the molecule concentration inside of the cell membrane (see also (2)). Therefore, the linear information-theoretic model described here is in accordance with biology and the way concentrations are measured at membrane transitions.  Section 2.4 will delve deeper into learning, but before continuing with learning, let us have a closer look at the formalization of Yin-Yang and how it relates to the linear information-theoretic model.

### 2.3. Yin-Yang

Duality is not an informal concept with little meaning outside the philosophical realm. On the contrary, this section will show that the high-level concept of Yin and Yang has a well-defined mathematical expression. In fact, Yin and Yang can be formalized with the mathematical, information-theoretic model introduced in Section 2.2, as a linear function of information. To do so, this section shows how the classic symbol of duality, namely, the Yin-Yang symbol, can be rendered using the linear information model. Note that the rendering described in the following is an improvement to the work presented in [3] in that the median distance between the “physical” Yin-Yang symbol and the rendered “information-theoretic” symbol is smaller.

**Figure 3:**
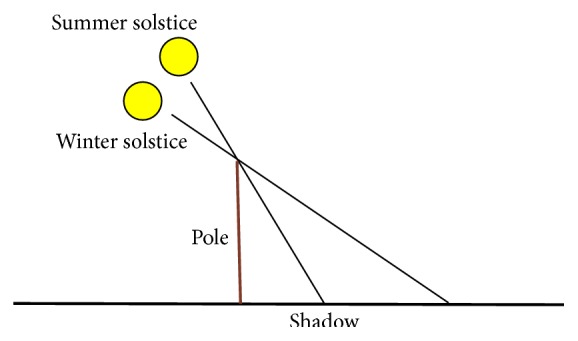
Yin-Yang daylight model [3].

According to the results in [3], the Yin-Yang symbol depicts the length of a pole's shadow when measured at the same time each day throughout the year, as symbolized in Figure 3. Plotting the number of daylight hours for the first half of the year and the number of hours of darkness for the second half of the year in a circular polar plot then produces the Yin-Yang symbol. In the following, the mathematics used to measure the number of daylight hours for each day of the year will be different from the one used in [3]. In particular, the model presented by Glarner in [10] will be used here. This model is clearer from a mathematical point of view in that it does not consider the light refraction in the atmosphere of the earth. The next paragraph follows the description in [10].

**Figure 4:**
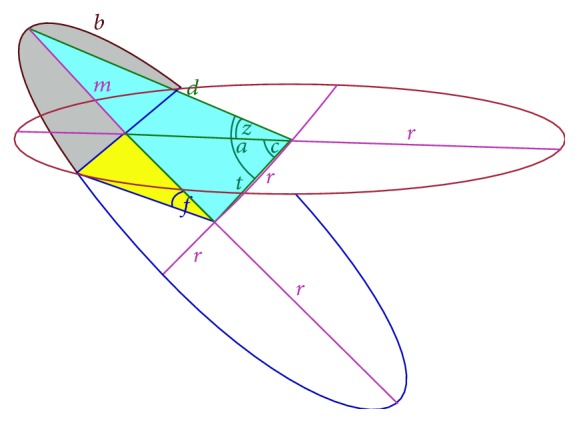
Solar circle for the summer solstice at 45° in the northern hemisphere (H. Glarner [10]).

The actual day of the year and the latitude of the observer both influence the length of the day. The perceived way of the sun around the planet can be viewed at as the boundary circle of the planet's disc. However, this constellation, in which the sun apparently circles along the disc's boundary, applies only at equinoxes and only at the North Pole. The further away the observer is from the North Pole (towards the equator), the more the surrounding circle is tilted along the west-east axis, until it is completely upright (perpendicular to the planet's disc) at the equator. Furthermore, there is also a shift of the circle away from the disc, along the obliquity of the ecliptic (connecting the centers of the two circles at an angle of 23.439°). This shift can be “upwards” (max. distance at the summer solstice) or “downwards” (max. distance at the winter solstice), depending on the actual latitude.  Figure 4 shows the tilted and shifted solar circle for the winter solstice at 45° North. It is only the part *b* out of the whole circle in which the sun in visible. When the sun is running along the blue part of the circle in Figure 4, it is night for the observer. The way to computing the number of daylight hours is now to calculate the exposed part *b* in relation to the whole circle [10]. The equations necessary to do so require three input parameters, namely,* Axis*,* Lat*, and* Day*:

(i) *Axis*. This is the obliquity of the ecliptic, which is the angle between the rotation axis of the earth and its orbital plane. The obliquity of the earth is about 23.4°. It can be considered a constant for the purpose of this paper because its values change only slowly over a period of thousands of years.
(ii) *Lat*. The latitude of the observer is in degrees. For example, for an observer at the equator,* Lat* is 0°. The latitude will increase for observers further north until it reaches 90° for an observer standing at the North Pole.
(iii) *Day*. This specifies the day of the year.* Day* runs from 0 to 364 for the first year, with 0.25 added from 365 for every completed year. Note that the day of year does not start with the astronomically quite arbitrary January 1st but with the day of the winter solstice in the first year of a four-year cycle [10].

Using these input parameters, computing the exposed fraction *b* of the sun's circle is a two-step process. First, the following intermediate result needs to be calculated:

(6) $$\begin{array}{c} m=1-\tan\left(Lat\right)\tan\left(Axis\cdot \cos\left(c\cdot Day\right)\right), \end{array}$$

where *c* ≈ 0.0172 is a constant. Note that the argument of the cos function is in radians, whereas the arguments of the tan functions are in degrees. The fraction *b* can then be computed as follows:

(7) $$\begin{array}{c} b=\frac{\arccos\left(1-m\right)}{180}. \end{array}$$

To get the number of hours the sun shines at the given* Day* and at the given Latitude* Lat*, *b* needs to be multiplied by 24. For a detailed derivation of these equations, readers are referred to [10].

Based on this computation of *b*, a linear regression function can be used to approximate the daily sunshine hours, as shown in [3]. For example, this produces the following approximation for one branch of the Yin-Yang symbol:

(8) $$\begin{array}{c} \Theta \left(p\right)=-3.208\cdot \log_{2}\left(p\right)+3.112. \end{array}$$

**Figure 5:**
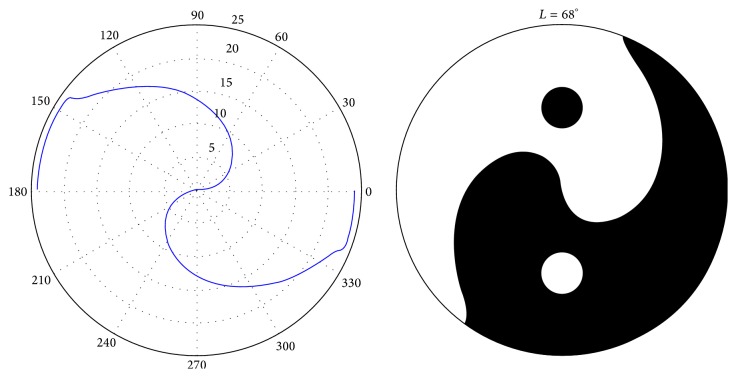
Yin-Yang symbol generated with the daylight model for *L* = 68° [3].

The median error for this branch of the Yin-Yang symbol is 0.08 h, which is less than half of the error reported in [3]. This result confirms again that the Yin-Yang symbol describes a linear information-theoretic function, as presented in Section 2.2. The left-hand side of Figure 5 shows the daylight/nighttime hours plotted into a polar plot for Latitude *L* = 68°. From the polar plot, the Yin-Yang symbol can be generated by rotating the plot by 90° and filling one area black and the other area white. The well-known dots of the Yin-Yang symbol are plotted halfway between the center of the circle and the circle's perimeter.

### 2.4. Learning

This section will have a closer look at learning. In particular, the linear learning equation in Section 2.2, (4), will be in the focus here. This equation provides two parameters that can be tuned for learning purposes: the slope *m* and the offset *c*. For the sake of simplicity, let us assume that the offset is constant and equals zero; that is, *c* = 0. The slope *m* then remains as the main parameter a synapse can learn. Furthermore, let us assume that the calcium ion concentration in the synaptic cleft close to the postsynaptic terminal, that is, *p*_*i*_, is the input that needs to be learned. This makes sense because *p*_*i*_ is directly affected by the input stimulus and the calcium ion concentration *p*_*i*_ can be considered as teaching input to the postsynaptic terminal, where learning takes place. The main learning task of a synapse then involves adjusting the slope *m* of the linear learning function until it matches the concentration *p*_*i*_; that is, *m* = *p*_*i*_. For the completed learning task, (4) can be written as follows:

(9) $$\begin{array}{c} I=-p_{i}\cdot \log_{2}\left(\frac{1-p_{i}}{p_{i}}\right). \end{array}$$

If we now require that the learned concentration *p*_*i*_, or slope parameter *m*, is equal to the input stimulus (1 − *p*_*i*_)/*p*_*i*_, then the following requirement needs to be satisfied [3]:

(10) $$\begin{array}{c} p_{i}=\frac{1-p_{i}}{p_{i}},\\ p_{i}=\frac{\sqrt{5}-1}{2}\;\text{or}\;p_{i}=\frac{-\sqrt{5}-1}{2},\\ p_{i}\approx 0.618\;\text{or}\;p_{i}\approx -1.618. \end{array}$$

This means that the learned concentration equals the input stimulus when the strength of the input stimulus matches the (reciprocal of) the Golden Ratio [11, 12]. As mentioned in [3], the results in the recent literature seem to indicate that the Golden Ratio plays a role in neural signal processing [13, 14]. This is another corroboration of the validity of the learning theory proposed here.

### 2.5. Dual Computation

The learning scheme represented by (9) describes the synaptic input-output relation after learning. However, the definition of input and output is arbitrary. In fact, the dual computation for which input and output change places is equally meaningful. To formulate a similar learning equation for the dual computation, (9) needs to be converted so that it applies in an antagonistic, symmetric way to the dual computation. This can be accomplished by transforming (9) into a symmetric form, under the assumption that the input signal (or stimulus) and the actually learned signal must be identical. Beginning with the linear learning equation, that is, Equation (9), the following transformations provide the desired symmetric form:

(11) I =−pi·log⁡21−pipi =−pi2·log⁡21−pi =−pi·log⁡21−pi2 =−pi·log⁡21−pi22.

**Figure 6:**
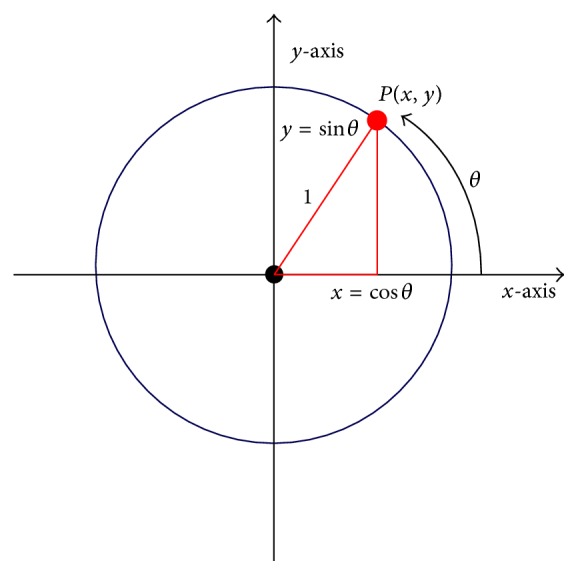
Perception points on the unit circle.

This can be written as follows:

(12) $$\begin{array}{c} \frac{I}{2}=-p_{i}\cdot \log_{2}\left(\sqrt{1-p_{i}^{2}}\right), \end{array}$$

which shows the symmetric relationship between the input signal and the learned output concentration. Note that *I* is multiplied by a scalar (1/2) in (12). This linear operation, however, only affects the scale of the learned information *I*. Because this affects *I* universally, it does not influence decision making. According to (12), for an input signal $\sqrt{1-p_{i}^{2}}$, the learned output is *p*_*i*_. Input and output thus define a point on the unit circle. The input can be considered the sine and the output the cosine of a point on the unit circle, as illustrated in Figure 6. All possible input-output combinations, or perceptual states, are points on the unit circle. Therefore, this relationship represents the desired symmetry between input and output. This symmetry allows to measure the input signal as *p*_*i*_ and the learned output signal as $\sqrt{1-p_{i}^{2}}$. For example, if the input signal is one, that is, the argument of the logarithm in (12) is one, then the learned output signal and uncertainty *I*/2 will be zero. However, because the dual computation behaves antagonistic and exchanges input and output, the output uncertainty of the dual computation will be infinite. Conversely, if the input signal is zero, then the learned output signal $\sqrt{1-p_{i}^{2}}$ is 1 and the corresponding output uncertainty *I*/2 is infinite. This means that the output uncertainty of the dual computation is zero. According to these results, the equilibrium state, in which both computations produce the same output uncertainty, is the state with the minimum overall uncertainty for both computations. Geometrically, this state is represented by a point on the unit circle for which both the sine and the cosine are $\sqrt{1/2}$. Therefore, sine and cosine can be considered Yin-Yang counterparts, and the output uncertainty *I* (or energy) is the Yin or Yang force, depending on the computation.

## 3. Results and Discussion

This section presents an application of the theoretical results derived above. For a lung screening application in which lung regions of chest X-rays are scanned for manifestations of tuberculosis, the machine confidence in abnormal regions will be graphically displayed.

### 3.1. Tuberculosis

Tuberculosis (TB) is the second leading cause of death from an infectious disease worldwide, after HIV, with a mortality rate of over 1.2 million people in 2010 [15]. With about one-third of the world's population having latent TB, and an estimated nine million new cases occurring every year, TB is a major global health problem. TB is an infectious disease caused by the bacillus Mycobacterium tuberculosis, which typically affects the lungs. It spreads through the air when people with active TB cough, sneeze, or otherwise expel infectious bacteria. TB is most prevalent in sub-Saharan Africa and Southeast Asia, where widespread poverty and malnutrition reduce resistance to the disease. Moreover, opportunistic infections in immunocompromised HIV/AIDS patients have exacerbated the problem. The increasing appearance of multidrug resistant TB has further created an urgent need for a cost-effective screening technology to monitor progress during treatment. A posteroanterior radiograph (X-ray) of a patient's chest is a mandatory part of every evaluation for TB [16, 17]. Therefore, a reliable screening system for TB detection using radiographs would be a critical step towards more powerful TB diagnostics. An automated approach for detecting TB manifestations in chest X-rays (CXRs) would allow cost-effective mass screening of large populations that could not be managed manually [1].

### 3.2. Computing Confidence

**Figure 7:**
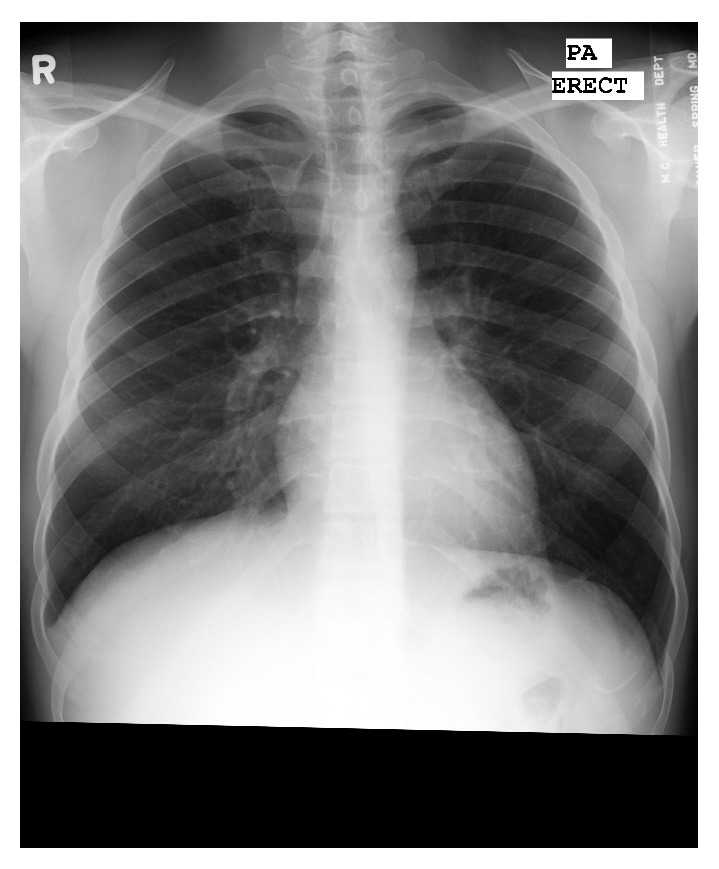
Normal chest X-ray (CXR).

**Figure 8:**
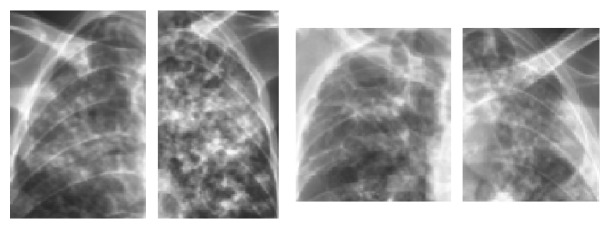
Abnormal lung patches identified by a radiologist.

As a step towards a fully automated system for TB screening in CXRs, and as an application example of the confidence values proposed here, this paper presents first experiments with a method for detecting manifestations of TB. The data being used is from Shenzhen No. 3 People's Hospital, China [18]. The CXRs were captured within a one-month period, mostly in September 2012, as part of the daily routine at Shenzhen No. 3 People's Hospital, using a Philips DR Digital Diagnost system. The data contains 342 abnormal images with manifestations of TB. For each image, a radiologist labeled the abnormal regions. Altogether 1671 regions have been annotated by two radiologists using the Firefly labeling tool [19], covering 18 different abnormalities, such as infiltrates, nodules, or effusions. For example, Figure 7 shows a normal chest X-ray, and Figure 8 shows a few samples of annotated abnormal lung patches. Each of the abnormal patches is represented by a set of histogram features that describe textures and shapes within the patch. In particular, the following features are used to describe the patch, each quantized into 32 bins: intensity, gradient magnitude, shape and curvature based on the Hessian eigenvalues, histogram of gradients, and local binary patterns [1]. All histograms are normalized and concatenated into a long 192-dimensional feature vector. To determine whether a region in a CXR is normal or shows a specific abnormality, the distance of the abnormal patches to the lung region can be computed by comparing the patch features with the features of the region. For the results presented here, the following histogram distance function is used to compute the distance between two normalized histograms *A* and *B*, where *A*_*i*_ and *B*_*i*_ denote the *i*th histogram bin, respectively, with ∑*A*_*i*_ = 1 and ∑*B*_*i*_ = 1:

(13) $$\begin{array}{c} D\left(A,B\right)=\frac{1}{2}\overset{N}{\underset{i=1}{\sum }}\left|A_{i}-B_{i}\right|. \end{array}$$

This is done for each feature, with *N* = 32, and the average distance among all features is computed as the distance between patch features and region features. To compute the confidence value and obtain the desired trigonometric relationship, the similarity between patch and lung region is computed as follows:

(14) $$\begin{array}{c} S\left(A,B\right)=\sqrt{1-D{\left(A,B\right)}^{2}}. \end{array}$$

*S*(*A*, *B*) is the confidence provided to the radiologist, indicating the machine confidence in the abnormality of the investigated lung region, given the presented patch.

### 3.3. Saliency Maps

**Figure 9:**
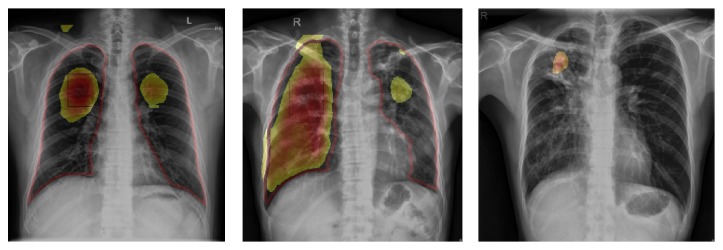
Saliency maps for X-rays from Shenzhen No. 3 People's Hospital.

By moving a known abnormal patch over a new input image and computing the similarity of the abnormal patch to the local lung region at each location, as described above, the entire input image can be screened for abnormal regions similar to the patch. When the confidence is recorded for each location, and different confidence values are displayed with different colors, a so-called saliency map can be generated. The saliency map highlights lung regions for which the machine is confident in their abnormality. For example, regions with high confidence can be marked in red, which provides direct feedback to the radiologist. The radiologist will be able to understand intuitively the different grades of confidence provided by the machine because the confidence is based on the sine/cosine relationship discussed above.  Figure 9 shows three examples of saliency maps computed for the Chinese X-rays. The first saliency map on the left-hand side shows an abnormality in the right lung (note that left/right are interchanged when describing lungs). The red color signifies that the machine is very confident that this region is indeed abnormal. The abnormality indicated in the left lung is less reddish, which shows that the machine is less confident that this is indeed an abnormal region. The red lung boundary is the result of an automated lung segmentation method [20]. The radiologist can ignore any machine confidence outside this region, if displayed at all. A similar case is shown in the middle saliency of Figure 9. In this example, the machine has confidence in the entire right lung being abnormal. Finally, in the third example, the machine has identified a relatively small region of abnormality in the upper right lung, with a relatively high confidence.

In these examples, the similarity function in (14) has been used to compute the confidence in the similarity of a lung region to a previously seen abnormal pattern. Alternatively, any classifier that outputs confidence values can be used for this task, such as the support vector machine used in [1] for discriminating between normal and abnormal lungs. Typically, confidence values are only considered when they exceed a threshold, which defines the operating point of the classifier and optimizes the classifier's sensitivity and specificity for a given cost function. For example, only dark red regions in the saliency maps in Figure 9 could be considered to reduce the false positive rate.

Note that the proposed method for representing confidence values is a postprocessing method. It can therefore be used in combination with any method that provides graded confidence in the similarity or dissimilarity of lung regions. In the lung screening application shown in this paper, the proposed method takes a dissimilarity measure of two lung regions and maps it to a similarity measure, according to the sine/cosine relationship. This would also work in the opposite direction, that is, mapping a similarity measure to a dissimilarity measure. The overall idea is that this sine/cosine relationship between similarity and dissimilarity is more intuitive, or natural, for human observers.

## 4. Conclusions

The paper proposes confidence values that can improve human-machine interaction for computer-aided diagnosis. The confidence values are motivated by neural signal processing and are intuitive in the senses that they are compatible with neural learning processes. Therefore, the operator of a computer-aided diagnostic system should be able to intuitively grasp the machine confidence and integrate it with his or her own confidence to reach a final diagnostic decision. The paper has revealed a direct connection to Chinese medicine and the dual concept of Yin-Yang, via a mathematical formalization of Yin and Yang. Furthermore, the paper shows that different learning states of a synapse, for which the input signal corresponds with the learned signal, can be represented by points on the unit circle. For the golden ratio, the learned signal corresponds with the actual signal. The state of equilibrium between Yin and Yang is the state for which sine and cosine are identical, which is the case at 45°. This state of equilibrium signifies neither normality nor abnormality and is thus a state of nonduality. As a practical example, the paper shows how the proposed confidence values can be used to highlight manifestations of tuberculosis in chest X-rays. In particular, the paper computes saliency maps where colors represent the magnitude of confidence values, indicating the confidence of the machine in the abnormality of a region in the chest X-ray. According to the theory set forth in the paper, the color intensities should be intuitive and the dynamic range of the colors (confidence values) should be similar to the representation a radiologist would use. As future work, the information-theoretic model presented in this paper could also help explain the efficacy of acupuncture in a more formal framework. For example, the paper explains Yin and Yang as energies of two dual computations performed at a synapse. Furthermore, the paper provides a well-defined mathematical definition of the state of equilibrium between Yin and Yang, which minimizes the overall energy for a synapse. A high energy in the input or output of a synapse could indicate an abnormal state, such as an inflammation for example. Acupuncture could bring such an abnormal state back into the equilibrium state, where information can flow freely, and where there is no excessive heat or cold at the input or output of a synapse.
